# Effects of the COVID-19 Pandemic on Psychological Well-Being and Mental Health Based on a German Online Survey

**DOI:** 10.3389/fpubh.2021.655083

**Published:** 2021-07-08

**Authors:** Katharina Lingelbach, Daniela Piechnik, Sabrina Gado, Doris Janssen, Martin Eichler, Leopold Hentschel, Dennis Knopf, Markus Schuler, Daniel Sernatinger, Matthias Peissner

**Affiliations:** ^1^Fraunhofer IAO, Fraunhofer Institute for Industrial Engineering IAO, Stuttgart, Germany; ^2^Department of Psychology, University of Oldenburg, Oldenburg, Germany; ^3^National Center for Tumor Diseases (NCT/UCC), University Hospital Dresden, Technical University Dresden, Dresden, Germany; ^4^seracom GmbH, Stuttgart, Germany; ^5^Department of Internal Medicine I, University Hospital Dresden, Technical University Dresden, Dresden, Germany

**Keywords:** psychological well-being, COVID-19 pandemic, mental and public health, mixed data clustering, correlation, multiple regression

## Abstract

**Objective and Background:** To contain the COVID-19 pandemic, public health actions have changed the everyday life with an inevitable impact on individuals and their social life. Since intact (socio-)psychological functioning and mental health are protective factors contributing to the immune system and preventing diseases, it is crucial to identify individuals with increased vulnerability.

**Methods:** We conducted a German online survey from April until August 2020 investigating health-related, social, behavioral, and psychological effects of the COVID-19 pandemic. One hundred and seventy three adults participating in the survey were analyzed (39.9% male, age: *M* = 44.81±13.31). We explored effects on mental health by (a) clustering participants in two clusters and (b) analyzing the clusters using correlations and regression models.

**Results:** Participants belonged either to a cluster characterized by higher general well-being or to a more concerned cluster depending on their responses. The correlation analysis revealed a significant negative relation between age and well-being with younger participants revealing higher depression scores in the concerned cluster. Furthermore, multiple regression models revealed that the number of risk factors only has a significant influence on psychological well-being in the concerned but not in the comfortable cluster.

**Conclusion:** We found that especially participants at (a) younger age and (b) greater risk of a severe course of disease reported reduced mental well-being and seemed to be weakened in their psychological protective factors in our sample. These insights allow to provide tailored recommendations for preventive and immediate actions to promote psychological well-being and reduce stress.

## 1. Introduction

The outbreak of the COVID-19 pandemic challenges not only the world-wide health and economic sectors (1) but also burdens individuals with concerns about their health due to a potential infection or infection of family members and friends as well as financial and occupational worries (2–6). Public health actions decreasing contacts to other persons might pay their toll and inhibit protective effects of social bonding and interactions. These social resources are important to cope with and alleviate psychological distress further promoting mental and physical health (7, 8). The impact of the COVID-19 pandemic on the mental health and protective resources might differ between individuals [e.g., due to preexisting psychological disorders, personality traits, or especially high concerns of persons at heightened risk of a severe course of disease (3, 9, 10); an occupation particularly burdened by the pandemic (11, 12); or due to insufficient coping strategies and resilience (9)]. Thus, it is important to identify especially vulnerable population subgroups and their characteristics in order to implement targeted preventive interventions promoting mental health without major delay.

Several already active longitudinal national and international surveys started investigating the impact of the COVID-19 pandemic on the social and psychological status with the advantage of a reference from former survey results (13–15). Pierce et al. (15) examined longitudinal changes in mental health from 53,351 adults in the UK using regression models. The authors reported an increase of 8.4% in the prevalence of clinically significant mental disorders from 2018/2019 to April 2020. In addition, values of the established General Health Questionnaire (GHQ-12) increased from 2018/2019 to April 2020 indicating more stress and lower mental health. The changes in mental health were particularly noticeable among women [cf., (16)], younger individuals between 18 and 34 years [cf., (14, 16, 17)], and persons living with young children (18). Another study investigated the psychological impact of COVID-19 cross-sectionally in China at an early stage of the pandemic using survey data from 1,210 participants (4). In total, 53.8% of participants rated the psychological impact of the pandemic outbreak as moderate or severe. 16.5% reported moderate to severe depressive symptoms, 28.8% moderate to severe anxiety symptoms, and 8.1% moderate to severe stress levels. In line with other literature (15, 19), they reported a greater psychological stress, anxiety, and depression in individuals with specific COVID-19 related physical symptoms and poor self-rated health status. Further risk factors for psychological stress include low socio-economic status, unemployment, and the frequent exposure to social media and news concerning COVID-19 [(18, 19), see (9, 20), for reviews]. Yamada et al. (21) conducted a large-scale cross-cultural study through a global survey (*N*=173,426) investigating the psychological (e.g., stress, trust) and behavioral responses (e.g., compliance) to the COVID-19 pandemic. Interestingly, the authors suggested a link between prolonged states of emergency and related stress with decreased compliance regarding public health actions for infection containment.

Further studies applied machine learning (ML) methods to examine the COVID-19 pandemic's psychological effects and relevant influencing factors. Analyzing posts in the Chinese online social network Weibo, Li et al. (22) observed increased negative emotions and sensitivity to social risks with decreased positive emotions and life satisfaction. Jha et al. (16) combined Bayesian networks and ML methods on a representative sample of 17,764 adults in the USA to identify key factors for mental health during the COVID-19 pandemic. They were able to predict the level of mental health of each individual with an average accuracy of ~80%. Based on these preliminary findings, especially women, younger participants, and participants at heightened risk of a severe course of disease should be identified as vulnerable in their psychological well-being (with increased anxiety, depression, and decreased quality of life) during the COVID-19 pandemic in the here investigated German sample. This should be reflected in their affiliation to the same cluster in a cluster analysis based on mental health with increased scores regarding anxiety, depression, and concerns as well as decreased quality of life.

To exploratory investigate effects of the COVID-19 pandemic on psychological well-being and mental health (here researched via scores describing anxiety, depression as well as quality of life), we applied an unsupervised clustering method suitable for mixed data sets (23) and analyzed the resulting clusters within correlations between age, sex, risk factors defined by the Robert-Koch Institute (RKI), infection concern, and contact behavior with several psychological scores, as well as regression models to identify the influencing key factors of those psychological scores.

## 2. Methods

We extracted the data from the still active German online survey WIBCE (German: “Was ich bei Corona erlebe”; English: “What I experience during Corona”) which started at the 1st of April 2020. Here, responses until the 24th of August from 173 participants were analyzed. Only the first entry of a participant and only those providing answers to all variables were included, which leads to a total number of 173 out of 275 participants (total number of drop-outs during the first participation: 260). Participants were recruited by convenience sampling via flyers and social media platforms and had to be of legal age. They could voluntarily answer the questions as many times as they liked even on a daily basis. However, some modules (e.g., items examining quality of life) were provided at a defined time interval (e.g., with at least a week in-between). Since answers were not mandatory, the number of provided responses differs for each item. The items comprise epidemiological and health-related information (COVID-19 specific, e.g., presence of symptoms, as well as general), the behavior in everyday life, social factors (e.g., number of contacted persons) as well as psychological factors (see Table 1 for detailed information).

**Table 1:** Overview of the variables of the online survey WIBCE.

**Variable name**	**Description of the variable**
**Variables used in the cluster analyses**	
Current well-being	5-point Likert scale: 0 (bad) to 4 (excellent)^*^
Occupational concern	4-point Likert scale: 1 (no concerns) to 4 (major concerns)^*^
Financial concern	5-point Likert scale: 1 (no concerns) to 4 (major concerns)^*^
Infection concern	5-point Likert scale: 1 (no concerns) to 4 (major concerns)^*^
Quality of life (EQ-5D-5L)	Sliding bar (continuous): 0 (low) to 1 (high)^**^
Depression (PHQ-2)	Sum score of two 4-point Likert scale items: 0 (no) to 6 (greater perceived depression)^*^
Anxiety (GAD-2)	Sum score of two 4-point Likert scale items: 0 (no) to 6 (greater perceived anxiety)^*^
**Additional variables used in the comparison, correlation, and regression models**	
Age	Years in numbers starting at 18^*^
Sex	Dummy variable: 0 (female) and 1 (male)
Psychological distress (PHQ-4)	Sum of the PHQ-2 and GAD-2: 0 (low) to 12 (high)^*^
Education	0, no school leaving certificate; 1, qualification after primary school;
	2, qualification after secondary school; 3, qualification after comprehensive secondary
	and specialized school, to 4 general qualification for university entrance^*^
Monthly net household income	0 (below 500€), 1 (501–1,000€), 2 (1,001–2,000€), 3 (2,001–3,000€), 4 (3,001–4,000€),
	5 (4,001–5,000€), 6 (5,001–6,000€), 7 (6,001–7,000€) to 8 (above 7,000€)^*^
COVID-19 risk factor for a severe infection	Dummy variable: 0 (no risk) and 1 (greater risk)
according to the Robert-Koch Institute (RKI)	
Sum of risk factors	Sum score of reported risk factors for one person^*^
Contact to others	Sum score with higher values indicating more active and frequent contacts with other people^*^
Multiple participation in WIBCE	Number of participation of a person in the survey since April 2020^*^

* *Step size = 1,*

** *Step size = 0.01. Risk factors defined by the RKI were listed in a information box for the respective items*.

To examine psychological well-being and mental health, we used the Patient Health Questionnaire-4 [PHQ-4; (24)] assessing psychological distress which includes the two dimensions (1) general anxiety [GAD-2; (25)] and (2) major depression [PHQ-2; (26)]. A study investigating the psychometric properties of the PHQ-4 in the German population with more than 5,000 participants reported good fit of the two-factor structure, good construct validity and acceptable internal consistency (27). In both, the PHQ-2 and GAD-2, participants had to judge the frequency of depression and anxiety within the last two weeks (“Not at all,” “Several days,” “More than half the days,” “Nearly every day”) by answering two questions each (PHQ-2: “Little interest or pleasure in doing things” and “Feeling down, depressed or hopeless”; GAD-2: “Feeling nervous, anxious, or on edge,” “Not being able to stop or control worrying”). Quality of life was examined via the examination of health-related quality of life [EQ-5D-5L; (28–30)]. Regarding the EQ-5D-5L, a systematic review by Buchholz investigating 24 studies describes it as a valid and reliable instrument with moderate to excellent reproducibility, good interrater-reliability and only minor ceiling effects (31). The questionnaire consists of five dimensions (1) mobility, (2) self-care, (3) usual activities, (4) pain/discomfort, and (5) anxiety/depression. Each dimension is measures with one item on a 5-level scale (“no problems,” “moderate problems,” “some problems,” “severe problems,” and “extreme problems”). Additionally, participants were asked to rate their current well-being, concerns regarding an SARS-CoV-2 infection, and future financial and occupational situation. To quantify participants' contact behavior, we calculated a score based on contact-related items (e.g., numbers of contacted persons in the last 24 h, contacts at work). Prior to the participation in the survey, participants agreed to an informed consent. The study was approved by the ethics committee of the Technical University of Dresden, Germany (EK-147042020).

### 2.1. Sample Description

The here analyzed sample comprised 173 participants (39.9% male, age: *M* = 44.81±13.31 and range: 18–74 years). On average, the household size was three (2.9) persons and 37.0% reporting to live with under-age children. 5.8% of the participants reported symptoms associated with COVID-19 and 40.5% were at higher risk of a severe course of disease. 79.8% graduated at least high school (here the German Abitur) reflecting a rather high educational level and 58.4% reported a monthly net household income of more than 3,000€.

### 2.2. Data Analysis

All data analyses were performed with custom written or adapted scripts in python^*TM*^ and IBM SPSS^Ⓡ^ 20.

#### 2.2.1. Cluster Analysis

To group the participants with similar characteristics regarding their psychological well-being and mental health in two subsets, the unsupervised clustering method Partitioning Around Medoids (PAM) with the k-Medoids algorithm (32) and the Gower distance as a similarity measure [(33); implemented in sklearn_extra (v.0.0.5) and sklearn (v.0.1.0b2) (34)] was used. This approach is especially suitable for mixed data sets comprising not only numerical variables (e.g., age) but also ordinal (i.e., scores) and nominal variables such as sex (23, 35–37). The Gower distance comprises several distance metrices, which are calculated depending on the respective type of data: For numerical and ordinal variables, the range-normalized Manhattan distance is typically used. Nominal variables are first dummy coded by assigning either a 0 or 1 for each category depending on the absence or presence of the qualitative attribute. In a next step, the Dice coefficient representing the distance between categories is calculated (33, 35). The individual distances between all variables of two elements are combined to the Gower distance. The matrix of the Gower distance contains the scores of all pair-wise element comparisons (33). We here defined the number of clusters in advance with *k* = 2 resulting in two clusters: one reflecting rather concerned participants with lower psychological well-being (*concerned*) and the other comfortable participants with less psychological distress (*comfortable*). The clusters were compared in their characteristics using 5,000-fold bootstrapped 95% confidence intervals (CI) of the mean value. No overlap between the CIs indicates a statistical significance of *p* < 0.01 and a partial overlap without including the means a statistical significance of *p* < 0.05 (38). In addition, we compared the bootstrapped cluster means with published reference values of the psychological variables GAD-2 (25), PHQ-2 (26), PHQ-4 (24), and EQ-5D-5L (28) before the outbreak of the COVID-19 pandemic.

#### 2.2.2. Correlations and Regressions

The focus of the following analyses lies on the identification of possible relations between the variables age, sex, contact behavior, concerns regarding an infection, risk factors, and the psychological scores for anxiety (GAD-2), depression (PHQ-2), psychological distress (PHQ-4, sum score of the GAD-2 and PHQ-2), and quality of life (EQ-5D-5L).

Due to non-normally distributed data, Spearman correlation (*r*_*S*_) analyses were performed for each of the two clusters concerned and comfortable, including only the first entry of each participant. The predefined significance threshold of *p* < 0.05 was adjusted for multiple comparisons via the Bonferroni correction.

To identify independent predictors of the psychological scores GAD-2, PHQ-2, PHQ-4 as well as EQ-5D-5L in both clusters, multiple linear regression models with the forward approach were used.

## 3. Results

### 3.1. Cluster Analysis

**Figure 1:**
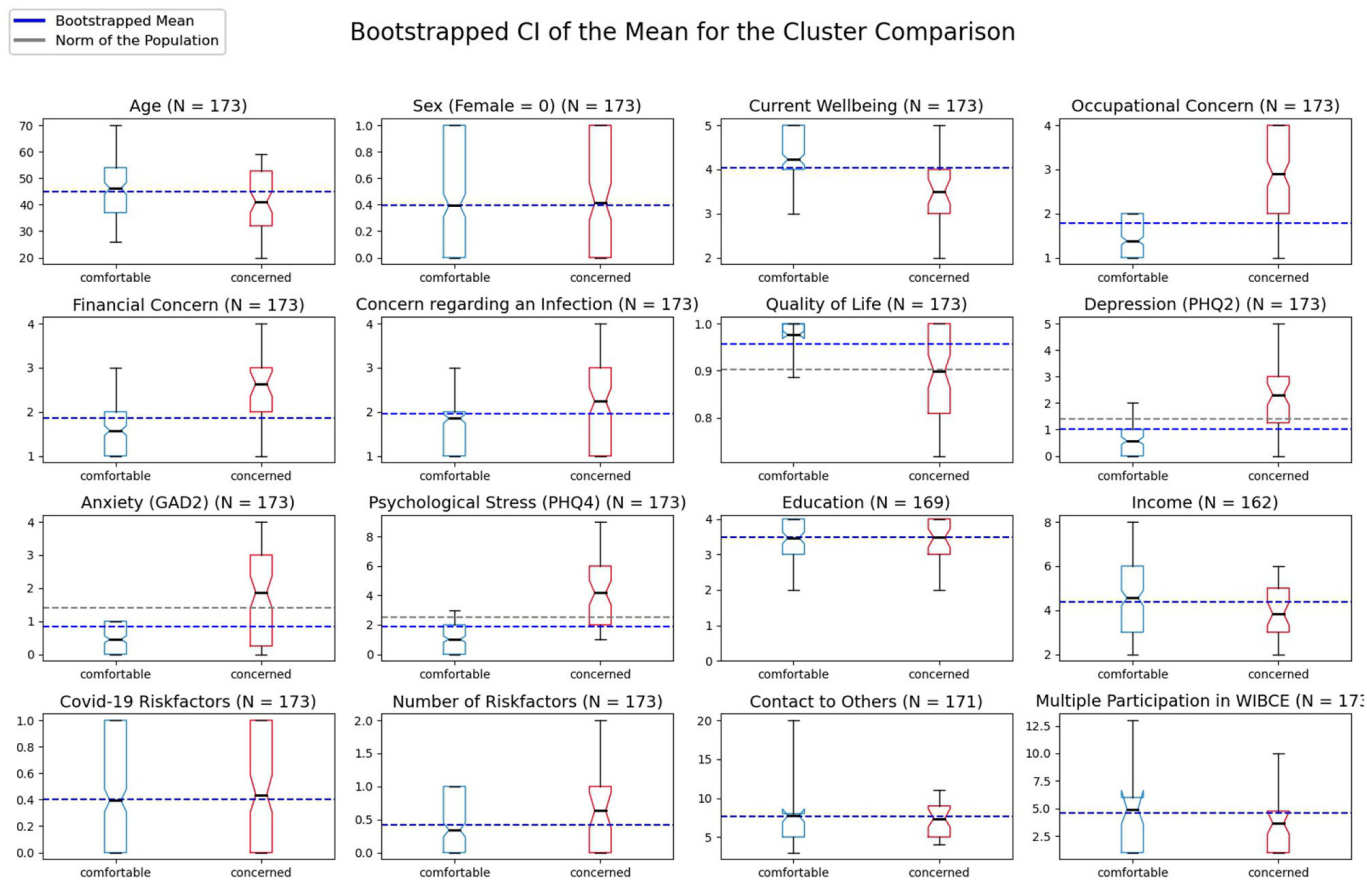
Bootstrapped means and 95% confidence intervals (CI) of the two clusters comfortable (blue) and concerned (red) for each variable. Notches in the boxes of the plot visualize the upper and lower boundary of the CI with the black small line in the box representing the mean of each cluster and the blue line representing the overall mean of the clusters. The gray line represents a population norm as reference with *M* = 0.902 for the EQ-5D-5L (28), *M* = 1.4 for the PHQ-2 (26), *M* = 1.4 for the GAD-2 (25), and *M* = 2.5 for the PHQ-4 (24). The box comprises 50% of the distribution from the 25th to the 75th quartile. The ends of the whiskers represent the 5th and 95th quartile of the distribution.

Using the k-Medoids clustering method, 127 participants were sorted into the comfortable cluster (39.4% male, age: *M*_*comfortable*_ = 46.17±12.68 years, 39.4% at greater risk of a severe course of disease) and 46 participants into the concerned cluster (41.3% male, age: *M*_*concerned*_ = 41.04±14.10 years, 43.5% at greater risk of a severe course of disease). When investigating the clusters' size as a function of first time participation, more of the participating persons belong to the comfortable cluster in early spring. While from May to August, participants were equally distributed to the clusters (see Supplementary Figure 1). Results of the cluster comparison for each variable with bootstrapped 95% CIs are provided in Figure 1 (also in further detail in the Supplementary Table 1). We observe a significant difference for age with *p* < 0.05 between the concerned (*M*_*concerned*_ = 41.06 [37.00, 45.17]) and comfortable (*M*_*comfortable*_ = 46.19 [43.91, 48.36]) cluster with more younger participants belonging to the concerned. However, the clusters do not differ in their distribution of sex. Regarding the risk of a severe course of disease, there is no difference between the clusters in the average presence of possible risk factors but a significant difference in the number of risk factors per person if present with higher numbers of risk factors in the concerned cluster (*M*_*concerned*_ = 0.63 [0.37, 0.91] vs. *M*_*comfortable*_ = 0.34 [0.24, 0.45]; *p* < 0.05). Interestingly, there is a significant difference in the variable income with lower monthly net household income in the concerned cluster (*M*_*concerned*_ = 3.84 [3.33, 4.40] vs. *M*_*comfortable*_ = 4.54 [4.20, 4.91]; *p* < 0.05). Being clustered on the psychological variables, the groups differ noticeably at *p* < 0.01 with higher values in the concerned cluster in their current well-being, occupational and financial concerns, concerns regarding an infection, depression (PHQ-2), general anxiety (GAD-2), and psychological distress (PHQ-4). Compared to reference values from the literature, the concerned cluster reveals significant higher depression [*M*_*concerned*_ = 2.30 [1.87, 2.72]; *M*_*Reference*_ = 1.4; (26)] and psychological distress scores [*M*_*concerned*_ = 4.17 [3.39, 5.02]; *M*_*Reference*_ = 2.5; (24)] but only slightly higher anxiety scores [non-significant (n.s.); *M*_*concerned*_ = 1.87 [1.39, 2.37]; *M*_*Reference*_ = 1.4; (25)]. The comfortable cluster is characterized by significantly lower anxiety (*M*_*comfortable*_ = 0.45 [0.35, 0.55]), depression (*M*_*comfortable*_ = 0.57 [0.43, 0.72]), and psychological distress (*M*_*comfortable*_ = 1.02 [0.83, 1.22]) compared to the reference values. Quality of life via the EQ-5D-5L was rated significantly lower in the concerned compared to the comfortable cluster (*M*_*concerned*_ = 0.90 [0.86, 0.93] vs. *M*_*comfortable*_ = 0.98 [0.97, 0.98]) but corresponds to the reference value *M*_*Reference*_ = 0.902 reported by (28). We investigated temporal cluster stability by clustering the last response of participants who participated at least twice in the survey (*N* = 90). 58.89% of those participants were clustered in the same cluster as before. 70.27% of the participants who changed clusters belonged to the concerned cluster at the second analysis. Only 29.73% of the participants changed from the concerned to the comfortable cluster.

### 3.2. Correlations and Regressions

To examine the relation between demographic, behavioral, and psychological variables, correlation analyses were performed for both of the clusters (Table 2).

**Table 2:** Results of the correlation analyses of age, sex, risk factors, infection concern, and contact behavior with several psychological scores (GAD-2, PHQ-2, PHQ-4, EQ-5D-5L) as well as the current well-being applying Spearman correlation (*r*_*S*_), subdivided into cluster concerned (a) and cluster comfortable (b).

	**Age**		**Sex**		**Risk factors**		**Infection concern**		**Contact behavior**	
	***r*_*S*_**	***p***	***r*_*S*_**	***p***	***r*_*S*_**	***p***	***r*_*S*_**	***p***	***r*_*S*_**	***p***
**(a) concerned cluster**										
Current well-being	−0.025	0.869	−0.111	0.463	−0.257	0.085	−0.339	0.021	0.186	0.22
GAD-2	−0.139	0.358	−0.247	0.098	0.239	0.11	0.378	0.009	−0.143	0.348
PHQ-2	−0.481^*^	0.001	−0.055	0.719	0.046	0.76	0.197	0.189	0.049	0.749
PHQ-4	−0.314	0.034	−0.177	0.24	0.16	0.287	0.387	0.007	−0.074	0.63
EQ-5D-5L	−0.089	0.553	0.127	0.401	−0.529^*^	0.001	−0.459^*^	0.001	0.175	0.25
**(b) comfortable cluster**										
Current well-being	−0.029	0.745	−0.034	0.707	0.016	0.86	0.102	0.253	−0.19	0.033
GAD-2	−0.274^*^	0.002	−0.067	0.452	−0.07	0.436	−0.014	0.872	0.141	0.114
PHQ-2	−0.216	0.015	−0.123	0.167	0.071	0.426	−0.143	0.110	−0.029	0.744
PHQ-4	−0.262	0.003	−0.122	0.173	−0.002	0.983	−0.093	0.301	0.026	0.774
EQ-5D-5L	−0.191	0.031	−0.094	0.292	−0.210	0.018	−0.052	0.561	0.069	0.446

* *p < 0.0025 (2-tailed, Bonferroni-corrected)*.

By analyzing possible relations between age and the psychological scores (GAD-2, PHQ-2, PHQ-4, and EQ-5D-5L) for the concerned cluster [Table 2(a)], a significant negative correlation is found for the major depression (PHQ-2; *r*_*S*_ = −0.481; *p* = 0.001) as well as a trend for the psychological distress score (PHQ-4; *r*_*S*_ = −0.314; *p* = 0.034), indicating that participants at younger ages show higher PHQ-2 and PHQ-4 scores (i.e., worse psychological well-being). In the concerned cluster, we observed a trend describing that a higher concern regarding an infection with SARS-CoV-2 also influences general anxiety (GAD-2; *r*_*S*_ = 0.378; *p* = 0.009; n.s.) and psychological distress (PHQ-4; *r*_*S*_ = 0.387; *p* = 0.007; n.s.). There is a significant negative correlation between concern regarding an infection and overall quality of life (*r*_*S*_ = −0.459; *p* = 0.001) in the concerned cluster. Interestingly, most of the psychological scores, except of the quality of life index (EQ-5D-5L; *r*_*S*_ = −0.529; *p* ≤ 0.001), seem to be not affected by the number of risk factors a participant has.

In case of the comfortable cluster [Table 2(b)], a significant negative correlation is found between age and the GAD-2 (*r*_*S*_ = −0.274; *p* = 0.002) as well as non-significant trends for the PHQ-2 (*r*_*S*_ = −0.216; *p* = 0.015), PHQ-4 (*r*_*S*_ = −0.262; *p* = 0.003), and EQ-5D-5L (*r*_*S*_ = −0.191; *p* = 0.031). In comparison to the concerned cluster, no significant relation was found between the concerns regarding an infection with SARS-CoV-2 and psychological well-being of participants. Interestingly, only in the comfortable cluster, a trend of a negative relationship between contact behavior and current well-being (*r*_*S*_ = −0.19; *p* = 0.033; n.s.) as well as the number of risk factors and quality of life (*r*_*S*_ = −0.210; *p* = 0.018; n.s.) exists. In both clusters no significant relation can be found between the participants' sex and their corresponding psychological scores.

To get a deeper understanding of what factors exactly influence psychological well-being, several multiple linear regression analyses were performed to identify possible predictors of the psychological scores GAD-2, PHQ-2, PHQ-4, and EQ-5D-5L based on a set of demographic, psychological, and behavioral variables, that are age, sex, risk factors, current well-being, infection concerns, and contact behavior. The analyses were performed for both clusters concerned and comfortable (Table 3).

**Table 3:** Possible predictors for GAD-2, PHQ-2, PHQ-4, and EQ-5D-5L scores, identified by multiple regression models using the forward approach, applied on both clusters concerned (a) and comfortable (b).

	**Dependent variables**							
	**GAD-2**		**PHQ-2**		**PHQ-4**		**EQ-5D-5L**	
**Variables**	***B***	***β***	***B***	***β***	***B***	***β***	***B***	***β***
**(a) concerned cluster**								
Age		−0.250	−0.057^*^	−0.559^*^	−0.106^*^	−0.528^*^		−0.019
Sex	−1.037	−0.302		0.195	−1.998	−0.346		−0.197
Risk factors		0.233	0.502	0.327	1.179^*^	0.392^*^	−0.067^*^	−0.533^*^
Current well-being		−0.232		−0.211	−0.767	−0.270	0.027	0.232
Infection concern	0.557	0.335		0.050		0.072	−0.029	−0.253
Contact behavior		−0.083		0.018		0.047		−0.063
*R*^2^	0.186		0.317		0.442		0.548	
adjusted *R*^2^	0.147		0.285		0.386		0.515	
**(b) comfortable cluster**								
Age	−0.013^*^	−0.280^*^	−0.015	−0.231	−0.028^*^	−0.306^*^	−0.001^*^	−0.249^*^
Sex		−0.079		−0.126		0.131		−0.097
Risk factors		−0.040		0.079		0.039		−0.170
Current well-being		−0.144	−0.331^*^	−0.273^*^	−0.455^*^	−0.265^*^		0.021
Infection concern		0.004		−0.089		−0.051		−0.065
Contact behavior		0.028		−0.146		−0.096		0.040
*R*^2^	0.079		0.123		0.158		0.062	
adjusted *R*^2^	0.071		0.109		0.144		0.054	

*The variance explained by all variables is given by R^2^, the variance explained only by the significant variables included in the model is given by the adjusted R^2^.*

* *p < 0.00625 (2-tailed, Bonferroni-corrected)*.

Table 3(a) summarizes the results of the linear regression models regarding the concerned cluster. The first regression model included infection concerns (β = 0.335; *p* = 0.021), followed by sex (β = −0.302; *p* = 0.036), as predictors for the general anxiety score (GAD-2). However, after correcting for multiple comparisons, there was only a trend observable indicating that the anxiety score increases 0.557 units for each unit of the score examining infection concerns and males have lower anxiety scores than females (−1.037 units). The major depression score (PHQ-2) decreases 0.057 units for each year of age and increases 0.502 units for increasing risk factors (n.s.). This is shown by the second model revealing the significant predictor age (β = −0.559; *p* ≤ 0.001), followed by a trend for the number of risk factors (β = 0.327; *p* = 0.018; n.s.). By examining possible predictors for psychological distress (PHQ-4), the third model revealed age (β = −0.528; *p* = 0.004), followed by risk factors (β = 0.392; *p* = 0.003), as significant predictors. Sex (β = −0.346; *p* = 0.026) and current well-being (β = −0.27; *p* = 0.019) were not significant after multiple comparison correction. As it was the case for the GAD-2 score, the PHQ-4 score decreases for each year of age (0.106 units) and increases for increasing risk factors (1.179 units) as well. Furthermore, there was a trend describing a decreasing PHQ-4 score with increasing current well-being (0.767 units, n.s.) and a trend for males reported lower psychological distress than females (−1.998 units, n.s.). On closer examination of the EQ-5D-5L, risk factors (β = −0.533; *p* = 0.001) was identified as significant predictor. Infection concerns (β = −0.253; *p* = 0.035) and current well-being (β = 0.232; *p* = 0.045) revealed only a trend after multiple comparison correction. Participants' quality of life decreases with increasing number of risk factors (−0.067 units) as well as increasing concerns regarding an infection with SARS-Cov-2 (−0.029 units; n.s.), and increases with higher current well-being (0.027 units; n.s.).

In case of the comfortable cluster [Table 3(b)], the linear regression models revealed age as a significant predictor for the psychological scores GAD-2, PHQ-4, and EQ-5D-5L. Similar to the concerned cluster, psychological well-being increases for each year of life, describing the fact that older participants are less affected by the psychological consequences of the COVID-19 pandemic. In case of the PHQ-2 and PHQ-4 score, current well-being was identified as significant predictor as well (PHQ-2: β = −0.273; *p* = 0.002; PHQ-4: β = −0.265; *p* = 0.002). In addition, those models show a smaller coefficient of multiple determination (adjusted *R*^2^), within a range of 0.054 (in the model describing the EQ-5D-5L score) and 0.144 (in the model describing the PHQ-4 score), than it is the case in the concerned cluster (here the adjusted *R*^2^ lies within a range of 0.515 in the model describing the EQ-5D-5L score).

## 4. Discussion

Within this study, we investigated the effects of the COVID-19 pandemic on psychological well-being and mental health by clustering participants into a concerned and comfortable cluster and analyzing the clusters with correlations and regression models.

In general, we observed significantly lower levels of mental health in the concerned than in the comfortable cluster. We found the same pattern for the reference values for depression (PHQ-2), anxiety (GAD-2; n.s.), and psychological distress (PHQ-4). Contrary to previous studies reporting a higher degree of psychological stress in women (16), women did not predominantly belong to the concerned cluster in our sample. However, the difference in comparison with other studies might be explained by the rather small sample size and selection bias due to convenience sampling. Although our results of the correlation analyses further suggest that sex is not significantly correlated with scores reflecting psychological well-being (in both clusters), there was a trend for sex as predictor in the regression model for general anxiety and psychological distress with higher values for women in the regression models of the concerned cluster (n.s.). Thus, it is likely that especially women are reporting low mental health and psychological well-being if they were identified as vulnerable and concerned via the cluster analysis. Regarding the influence of the variable age, more younger participants belonged to the cluster with lower psychological well-being and mental health. The correlation analyses additionally support the assumption that participants at younger ages show worse psychological well-being not only in the concerned but also in the comfortable cluster. Moreover, it is a significant predictor for depression (PHQ-2) and psychological distress (PHQ-4) in the regression models. Although, age is a significant predictor in the regression models of both clusters, the effect is much smaller in the comfortable compared to the concerned cluster. In addition, the predictor current well-being with higher values indicating reduced depression and psychological distress was only significant in the comfortable cluster. The variables sex, risk factors, infection concern, and contact behavior do not provide any added value for the explanation of psychological well-being and were, therefore, excluded from the models. We assume that some of those variables like infection concern could already be included in current well-being, since concern of an infection strongly influences current well-being of the participants. In accordance with (39), our results suggested that older participants are less affected in their psychological well-being by the consequences of the COVID-19 pandemic as reflected in a significantly higher average age in the comfortable cluster and smaller effect of age in the corresponding regression models. The clusters did not differ in the presence of risk factors leading to a severe course of disease. However, when risk factors were present, the concerned clusters reported on average a higher number of risk factors. Although the number of present risk factors is not significantly correlated with psychological well-being in both clusters, it was a significant predictor for psychological distress (PHQ-4) and quality of life (EQ-5D-5L) in the regression models of the concerned cluster. Interestingly, we observed rather small effects in the models predicting quality of life (EQ-5D-5L) in comparison with the other scores. The bootstrapped mean score of the comfortable cluster was even significantly above the reference values and the concerned cluster revealed comparable mean quality of life. Our finding of a significant difference regarding the average income between the concerned cluster and comfortable cluster is in line with (18) and (19) reporting also a negative correlation between income and mental health. However, the lower income in the concerned cluster might also be explained by the fact that it contained more younger people which are rather at the beginning of a career or still in their education without regular income. The clusters did not differ in their social behavior (i.e., number of contacted persons). Hence, either the reduced contact to other persons due to social distancing actions did not have a strong influence on psychological health and could not fully explain differences in psychological health between the clusters; or the concerned cluster suffered particularly strongly due to the social distancing. Since we have no reference value before the COVID-19 pandemic for the number of contacted persons of the two clusters, it is also possible that the concerned cluster comprising rather younger persons typically cultivating larger peer-group friendships reduced their social contacts significantly more compared to the comfortable cluster. In case of a strong impact on the psychological well-being and mental health of the concerned cluster due to social distancing actions, countermeasures fostering social activity in accordance with COVID-19 protection measures should be extensively explored in the future (e.g., virtual platforms and events especially with a focus on nearby regional areas). Thereby, such countermeasures should be tailored to the interests and needs of the concerned cluster. Our results suggest that socioeconomic factors play an important role in the emotional management of the pandemic. These findings should be considered and integrated by physicians and therapists. Regarding limitations of the study, there might be a selection bias in our sample, since Internet use and access is not equally distributed across age groups and socioeconomic groups. In addition, individuals with higher socioeconomic status more likely participate in surveys using convenience sampling (40, 41). Furthermore, it is not known how the special conditions of the pandemic affected the willingness and ability to participate and whether this influence is equally distributed among the population. Hence, it might be that people working from home or on short-time work had higher participation rates. Considering power and sample size, Formann (42) suggests a minimal sample size of 2^*k*^ samples with k reflecting the number of variables used in a cluster analysis. Although our sample size exceeds this suggestion, a larger sample with 5*(2^*k*^) (42) as rule of thumb would be beneficial to ensure stability and representativity of the clusters. An additional limitation of our study is the missing information of psychological well-being and mental health conditions of the participants before the outbreak of the pandemic. The described results do not take into account the history of mental health problems before the pandemic. Longitudinal active surveys should investigate the here suggested approach to explore effects of COVID-19 in a pre-post-design and with a more representative sample reflecting the general population. Further unknown confounding factors cannot be ruled out in this observational study. A preliminary analysis of cluster stability based on participants who provided at least two responses revealed a profound influence of time on the cluster affiliation. Especially in times of crisis, as in the case of a pandemic, it is likely that protective resources and coping abilities will be exhausted over time. In a next step, we plan to further analyze the longitudinal data of the German sample to investigate the temporal stability of psychological well-being and potential influences within the clusters.

## 5. Conclusion

Our study replicated previously reported results [e.g., (4, 9, 16–18)] of lower psychological well-being and mental health during the COVID-19 pandemic in (i) women weakened in their psychological factors (supported by the regression analyses of the concerned cluster), (ii) persons weakened in their psychological factors and with higher risk factors, and (iii) younger persons. Our approach could identify individuals vulnerable in their psychological well-being and mental health and key factors influencing the psychological well-being during the COVID-19 pandemic. The machine learning approach has the ability to learn rules which identify complex relationships between the variables in large datasets. Thereby, it integrates the information of several established scores to detect concealed patterns in the data. The results allow to provide individual and cluster-tailored recommendations in a clinical and everyday context [e.g., (43)] for preventive and immediate actions potentially helping to alleviate concerns, decrease perceived psychological distress and foster psychological well-being as well as quality of life.

## Data Availability Statement

The raw data supporting the conclusions of this article will be made available by the authors, without undue reservation.

## Ethics Statement

This study was approved by the ethics committee of the Technical University of Dresden, Germany (EK-147042020). The patients/participants provided their written informed consent to participate in this study.

## Author Contributions

KL, DJ, ME, DK, LH, MS, DS, and MP planned the research. The survey was developed by ME, LH, MS, DK, and DS. DK and DS provided the technical infrastructure. Data analysis was done by KL, SG, and DP. Cluster analysis was done by KL and SG. Time series analysis was done by DJ and SG. The interpretation was carried out by DP, KL, SG, and DJ. KL, DP, SG, DJ, ME, LH, and MP prepared the manuscript. All authors contributed to the article and approved the submitted version.

## Conflict of Interest

DK and DS were employed by the company seracom GmbH. The remaining authors declare that the research was conducted in the absence of any commercial or financial relationships that could be construed as a potential conflict of interest.
