# Supplementary material for: Effects of the COVID-19 Pandemic on Psychological Well-Being and Mental Health Based on a German Online Survey
Source: Front Public Health. 2021 Jul 8;9:655083. doi: 10.3389/fpubh.2021.655083 (PMC8296300; doi:10.3389/fpubh.2021.655083)
Supplement: Supplementary file 1 [file Data_Sheet_1.PDF]

SUPPLEMENTARY MATERIAL

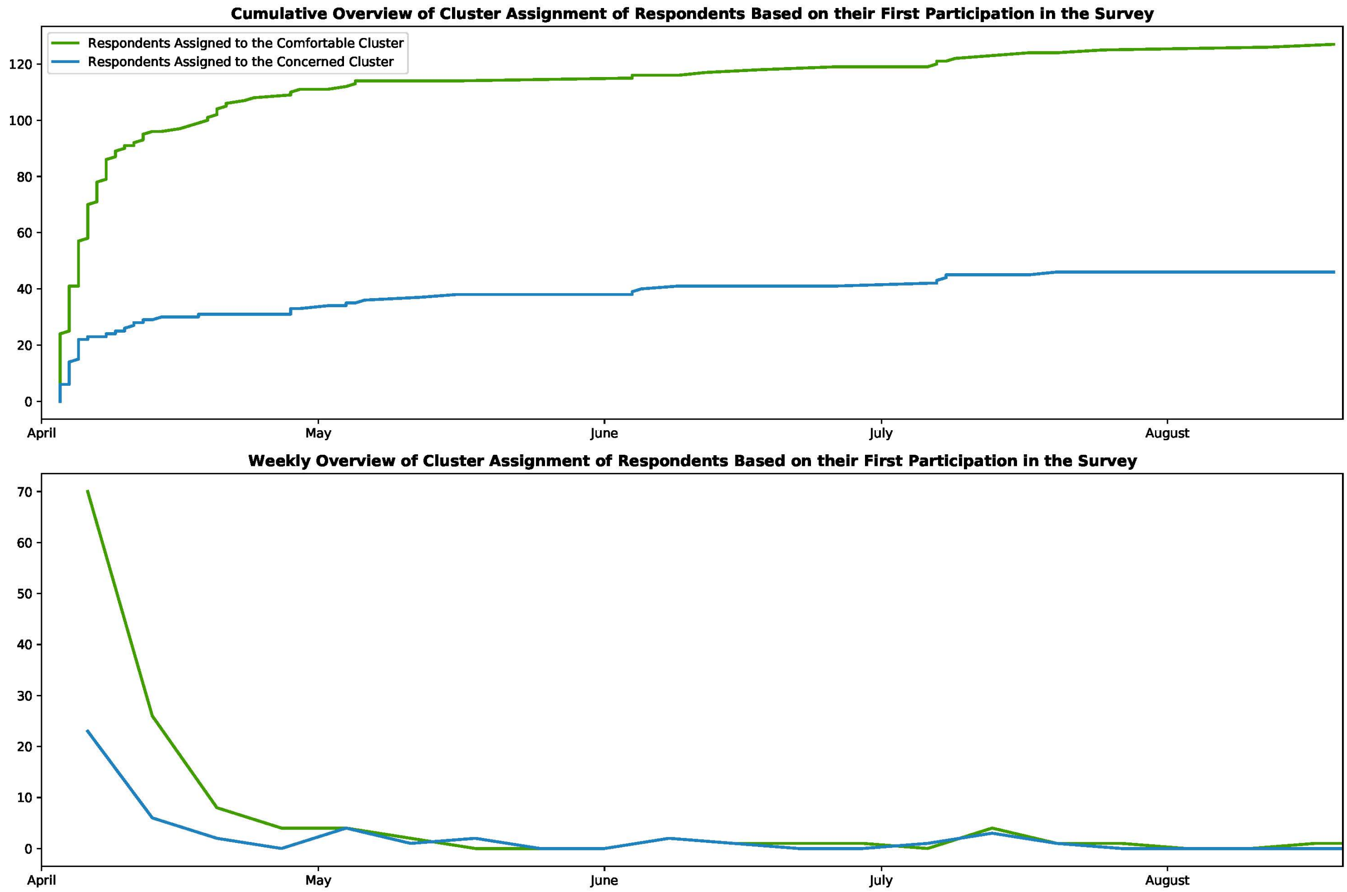

Figure 1. Cumulative number of respondents assigned to the clusters over the months. Only the first participation per person was taken into account.

Table 1. Bootstrapped means and 95 % CI with the lower and upper bound for the features and clusters.* significant at $p < .05$. ** significant at $p < .01$.

Variable Name	Cluster Comfortable	Cluster Concerned
Age*	46.19 [43.91, 48.36]	41.06 [37.00, 45.17]
Sex	0.39 [0.31, 0.48]	0.41 [0.28, 0.57]
Current well-being**	4.23 [4.11, 4.34]	3.50 [3.20, 3.78]
Occupational concerns**	1.38 [1.29, 1.46]	2.89 [2.61, 3.17]
Financial concerns**	1.57 [1.46, 1.69]	2.63 [2.35, 2.89]
Concerns regarding an infection**	1.85 [1.74, 1.95]	2.24 [1.96, 2.54]
Quality of life (EQ-5D-5L)**	0.98 [0.97, 0.98]	0.90 [0.86, 0.93]
Depression (PHQ-2)**	0.57 [0.43, 0.72]	2.30 [1.87, 2.72]
Anxiety (GAD-2)**	0.45 [0.35, 0.55]	1.87 [1.39, 2.37]
Psychological Distress (PHQ-4)**	1.02 [0.83, 1.22]	4.17 [3.39, 5.02]
Education	3.47 [3.31, 3.63]	3.48 [3.20, 3.72]
Income*	4.54 [4.20, 4.91]	3.84 [3.33, 4.40]
COVID-19 risk factor	0.39 [0.31, 0.48]	0.43 [0.30, 0.57]
Numbers of risk factors*	0.34 [0.24, 0.45]	0.63 [0.37, 0.91]
Contact to Others	7.71 [6.94, 8.56]	7.35 [6.27, 8.60]
Multiple Participation in WIBCE*	4.92 [3.62, 6.60]	3.65 [2.70, 4.74]
